# Recent Advances in Endocannabinoid System Targeting for Improved Specificity: Strategic Approaches to Targeted Drug Delivery

**DOI:** 10.3390/ijms232113223

**Published:** 2022-10-30

**Authors:** Mendhi Henna Dasram, Roderick B. Walker, Sandile M. Khamanga

**Affiliations:** Division of Pharmaceutics, Faculty of Pharmacy, Rhodes University, Makhanda 6139, South Africa

**Keywords:** endocannabinoid system, receptor-mediated drug delivery, endocannabinoid tone, g-protein coupled receptors, allosteric modulation, biased signaling, surface-modified nanoparticles

## Abstract

Opportunities for developing innovative and intelligent drug delivery technologies by targeting the endocannabinoid system are becoming more apparent. This review provides an overview of strategies to develop targeted drug delivery using the endocannabinoid system (ECS). Recent advances in endocannabinoid system targeting showcase enhanced pharmaceutical therapy specificity while minimizing undesirable side effects and overcoming formulation challenges associated with cannabinoids. This review identifies advances in targeted drug delivery technologies that may permit access to the full pharmacotherapeutic potential of the ECS. The design of optimized nanocarriers that target specific tissues can be improved by understanding the nature of the signaling pathways, distribution in the mammalian body, receptor structure, and enzymatic degradation of the ECS. A closer look at ligand-receptor complexes, endocannabinoid tone, tissue distribution, and G-protein activity leads to a better understanding of the potential of the ECS toolkit for therapeutics. The signal transduction pathways examine the modulation of downstream effector proteins, desensitization, signaling cascades, and biased signaling. An in-depth and overall view of the targeted system is achieved through homology modeling where mutagenesis and ligand binding examine the binding site and allow sequence analysis and the formation of libraries for molecular docking and molecular dynamic simulations. Internalization routes exploring receptor-mediated endocytosis and lipid rafts are also considered for explicit signaling. Furthermore, the review highlights nanotechnology and surface modification aspects as a possible future approach for specific targeting.

## 1. Introduction

The discovery of the endocannabinoid system (ECS) began with the isolation of the active ingredient in an extract of *Cannabis sativa*, observation of endogenous ligand binding, the genetic and physical mapping of the human cannabinoid receptor gene, and identification of enzymes that synthesize and degrade endocannabinoids and has resulted in increased interest and global acceptance of the use of *Cannabis sativa* [1]. The growth in clinical evidence for the therapeutic efficacy of cannabinoid formulations can be attributed to knowledge of molecular genetics of cannabis, successful decoding of the genome, and crystal structure determination of cannabinoid receptor type 1 (CB1-R) and cannabinoid receptor type 2 (CB2-R) with their heterotrimeric complex formation [2] [NO_PRINTED_FORM]. This paper gives an overview of knowledge of the ECS to provide a roadmap for navigating ECS targeted drug delivery [3,4,5,6,7]. Reviews of the discovery and operation of the ECS, pharmacotherapeutics of the ECS and optimization of nanomedicine for targeted drug delivery have been published [8,9,10,11,12,13,14]. The purpose of this review is to examine recent advances in ECS targeting for improved specificity and highlight strategies that have been used to improve nano drug delivery systems for targeting this system.

The global trend of decriminalizing *Cannabis sativa* is not without controversy; and the legality and stigma associated with the plant slowed the transition of use into the pharmaceutical industry [15]. Challenges associated with pharmacokinetics, formulation of highly lipophilic cannabinoids, psychoactive and off-target effects, and high-dose dependency associated with delta-9-tetrahydrocannabinol (Δ9-THC) have further hindered the commercialization of cannabinoid-containing products [16]. Direct activation of CB1-R is responsible for most associated adverse effects such as hypomotility, hypothermia, and catalepsy [17]. CB1-R activation can also induce sedation, hyperphagia, and physical or mental dependence [18]. While the desired anti-inflammatory and immunosuppressive properties of CB2-R activation in neurons are important and often sought, the peripheral CB2-R activation in T-cells which reduces immune response may be unfavorable in pathogen-associated inflammation or cancer [19,20,21]. In an attempt to avoid direct activation of cannabinoid receptors, studies involving alteration in endocannabinoid tone by fatty acid amide hydrolase (FAAH) and monoacylglycerol lipase (MAGL) inhibitors have yielded some promising preclinical results but have been unsuccessful thereafter [22].

The most promising receptors and molecular targets in the endocannabinoid system have been identified which suggests strategic targeting approaches may improve drug delivery [23]. The literature is replete with target sites in the body and activate or inhibit signal transduction pathways with endocannabinoid (EC) degrading enzyme inhibitors, EC uptake inhibitors, upregulated cannabinoid receptors (CB-R) in pathophysiological states, cannabimimetic compounds and other drugs which when taken concurrently with cannabinoids, enhance the therapeutic properties of cannabinoids [24,25]. Following crystal structure elucidation CB-R was confirmed to be a seven-transmembrane G-protein coupled receptor (GPCR) [26]. The wealth of knowledge relating to GPCR mediated drugs has been translated to commercial products and are important to consider when developing targeted delivery to CB-R [27]. GPCR are the largest class of cell surface receptors which make up approximately one-third of currently marketed drugs [28]. GPCR are involved in signaling pathways that control all essential processes, including vision and carcinogenesis, making them viable options for therapeutic intervention [29]. An understanding of signal transduction pathways following the orthosteric, biased or allosteric binding of ligands and intracellular modulators to the CB-R-G-Protein complex has been identified as a viable strategy for specific targeting [30]. With the aid of homology and modelling drug concentration at the receptor site and the intensity of a drug effect can be elucidated [31]. However, receptor density on the cell surface may influence the drug-receptor response, signal transmission mechanism into the cell by secondary messengers, or regulatory factors that control gene translation and protein production [32,33,34]. While CB1-R and CB2-R mediate the function of multiple tissues, glands, and organs in the body, their expression varies throughout the body [35]. The location and distribution of CB receptors modulate their physiological effects therefore designing drug delivery systems for bio-specific targeting may be achieved when homology and modeling are used to precisely map the subcellular, cellular, tissue, and regional distribution of cannabinoid receptors. CB1-R is highly expressed in the central nervous system but is only present in the peripheral tissues in lower amounts, whereas CB2-R is expressed primarily in immune cells, with varying levels found throughout the body including the brain, liver, myocardium, and coronary endothelial and smooth muscle cells [36]. CB2-R has been found in the adrenal glands, myocardium, endothelium, testis, uterus, bone, prostate, gut, smooth muscle of the vasculature and in different tumors [37,38,39]. The homology of the two receptors is structurally different, allowing the development of subtype-selective ligands for more specific targeting [40,41].

The strategies used to target sites where the endocannabinoid system is already active by enhancing endogenous cannabinoid tone appear to be more selective with minimal side effects and directly activate cannabinoid receptors [42,43]. However, the rationale for improving receptor-mediated drug delivery using nano precision tools which include nanocarriers properties consideration of factors such as size, shape, elasticity, surface charge and interaction of materials with the immune system, blood, plasma, cell membranes and biological barriers have been reported [44,45]. Surface modification by changing ligand surface density and surface patterning can affect cellular internalization and binding affinity [46,47]. To date, cannabinoid receptors have been identified and classified based on their structure, ligand-binding properties and signaling system used, and the cDNA and genomic sequences encoding CB receptors (*Cnrs*) from several species have been cloned. In this review, we describe the use of different canonical and non-canonical receptors, phytocannabinoids, natural and synthetic endocannabinoids used to achieve specific drug delivery targeting.

## 2. The Endocannabinoid System (ECS)

### 2.1. The History of Cannabis

*Cannabis sativa* is the most widely used illicit recreational and medicinal drug, in modern Western Society. Cannabis also has the most extended history of human use recorded compared to any other illicit drugs. The popularity of use of the plant lies in an ability to alter sensory perception and cause elation and euphoria [48]. The French poet, Charles Baudelaire, in his book, “Les Paradis Artificiels” published in 1860, describes being under the influence of hashish as ‘the playground of seraphim’ and a little green sweetmeat’ [49].

*Cannabis sativa* has been used as a fibre plant, a grain crop, and a medicinal plant in China since 4000 BC [50]. The fibres from cannabis stems were used to manufacture strings, ropes, textiles, and paper, and evidence of hemp-woven textiles and paper were found in the tomb of Emperor Wuof the Han Dynasty (104–87 BC). There has been a continuous recorded history of cannabis cultivation from Neolithic times down to the present [51]. According to the dynastic history Hou Han Shu, a famous physician Hua T’o (117–207 AD), used a concoction of cannabis called ma-fei-san (hemp-boiling-compound) taken with wine to anaesthetise his patients in order to perform operations on the abdominal organs [52]. The effect of temporal distortion or hallucinations caused by cannabis was noted by others [53]. The *Cannabis sativa* plant used as a medicine by the ancient Chinese civilization was reported in the oldest known Chinese pharmacopoeia, the Shen-Nung Pen-ts’ao Ching, which lists 366 herbs and 1000 herbal formulations [54,55]. Shen-nung Pen-ts’ao Ching is a compiled compendium of materia medica, pharmacology, and pharmacopoeia formally published as the first official materia medica in Chinese history [56]. In these herbal products, cannabis was used as a cure for many diseases, including malaria, constipation, disorders associated with the female reproductive system, rheumatic pain, and others [57].

Many prescriptions were filled for the analgesic effect, especially for severe pain due to broken bones. Cannabis seeds are still prescribed as a laxative by Chinese physicians today [58]. The morphology of the cannabis fruit, and the layers which cover it, are misunderstood, and is generally inadequately described [59]. Cannabis seeds have a low Δ9-THC content and are made up of essential fatty acids and proteins of which fourteen fatty acids have been identified in different cannabis seed preparations [60]. In ancient India, cannabis was integrated into religious and medical practices around 1000 years BC [61]. *Cannabis indica* is mentioned in many ancient sacred Indian scriptures and folklore. The Indian Hemp Drugs Commission of 1893–1894, following testimony from hundreds of native and Western doctors about the therapeutic uses of cannabis [62] concluded the following, that the hemp drugs used in modern European therapeutics appear to be used for the same purposes and precisely the same manner as it was recommended by native Indian doctors centuries ago, and suggested that *Cannabis indica* is one of the most important drugs of Indian material medica [63].

Cannabis as a psychoactive substance reached Europe and America through trade with the Arab community in the 19th century to 1937, when it was banned in the United States of America [64]. The isolation and elucidation of plant-derived phytocannabinoids offered new insights into the mechanisms underlying the therapeutic activity of cannabinoids and novel molecular targets for pharmacotherapy [65]. The identification of the chemical structure of the components of cannabis made it possible to isolate pure constituents and which significantly increased scientific interest in the cannabis plant from the 1960s. A further spike in interest occurred in the 1990s when the description of cannabinoid receptors and the discovery of the internal endocannabinoid system was reported [51]. The WHO recommended the rescheduling of Cannabis which was adopted by the United Nations’ Commission for Narcotic Drugs in December 2020 [66,67]. There is certainty in the therapeutic benefits of *Cannabis sativa*, although limited by the high concentration of Δ9-THC compounds which dominates the present-day *Cannabis sativa* plant due to the changes in cannabis potency over the last few decades [68]. The last few decades have seen exponential growth in cannabis sativa research at a higher rate than any time in the thousands of years that cannabis has been used by humans, and particularly in cannabinoid genomics [69].

### 2.2. The Disovery of the Endocannabinoid System

The ECS is a neuromodulatory system found throughout the human body [70]. In 1992, a cannabinoid receptor binding study identified the existence of an endogenous ligand for cannabinoid receptors and the structure thereof elucidated with mass spectrometry and nuclear magnetic resonance (NMR) spectroscopy. The natural anandamide was named [71]. Identifying anandamide as an endogenous ligand reaffirmed the biological importance of fatty acid amides and derivatives reported previously [72]. An NMR spectrum of synthetic palmitoylethanolamide exhibited similar chemical shifts and spin-coupling patterns to that of anandamide [71]. Prior to identifying the ECS, the biological attributes of anandamide were considered due to fatty acid amides and their derivatives. In 1957, the anti-inflammatory effects of palmitoylethanolamide from egg yolk were documented [73] and in 1990 the angiogenic effects of a fatty acid amide located in the bovine mesentery was reported [74]. Synthetic arachidonamide was found to inhibit leukotriene biosynthesis [75] which led to the thinking that anandamide is formed via arachidonic acid metabolism forming compounds that act on cannabinoid receptors, resulting in the conceptualization of an endogenous cannabinoid system [76]. The ECS is comprised of endogenous lipid cannabinoids termed endocannabinoids, cannabinoid receptors (CB-R) and enzymes that synthesize and degrade endocannabinoid compounds. Endocannabinoids precursors present in lipid membranes undergo enzymatic catabolism to form endocannabinoids. Upon activation of G-protein-coupled receptors, endocannabinoids progress in enzymatic steps following release into the extracellular space [76]. Endocannabinoids are potent regulators of synaptic function throughout the central nervous system, with the primary mechanism of regulation of retrograde signaling [77]. The decrease in cAMP in neuroblastoma cell cultures following administration of cannabinoids provided evidence that confirmed the existence of cannabinoid receptors mediated by G*_i/o_*-coupled receptors [78].

### 2.3. Cannabinoid Receptors

Any biological macromolecule that may bind a drug and generate a measurable response, is considered a receptor. Two important properties of receptors are binding according to the laws of thermodynamics which is typically stereo-selective, saturable, and reversible in nature or signal transduction*,* which follows a transduced functional response which is biological or physiological following the commencement of binding. Typically, a receptor has two main domains, a ligand-binding, and an effector domain. Receptor overexpression, unique binding motifs, and receptor patterning on the cell surface must be considered when selecting a receptor for receptor-mediated drug delivery technologies [79].

The isolation and sequencing of the superfamily, seven-transmembrane (7TM) G-coupled protein receptors commenced with the successful cloning of the $\beta_{2}\;$ adrenergic receptor [80]. This discovery acted as a model system for future studies of membrane proteins and approximately one thousand G-coupled protein receptor sequences have been identified, and four hundred receptors have been mapped throughout the body including rhodopsin, olfactory, adrenergic, the peptide hormone glutamate, and neurotransmitter gamma-aminobutyric acid (GABA) receptors [81]. The six hundred remaining receptor sequences were grouped as ‘Orphan Receptors’ as they could not be classified into any of the aforementioned subcategories. This significant discovery provided an opportunity for potential new developmental drugs, as new possible target receptors were being elucidated [82].

**Figure 1:**
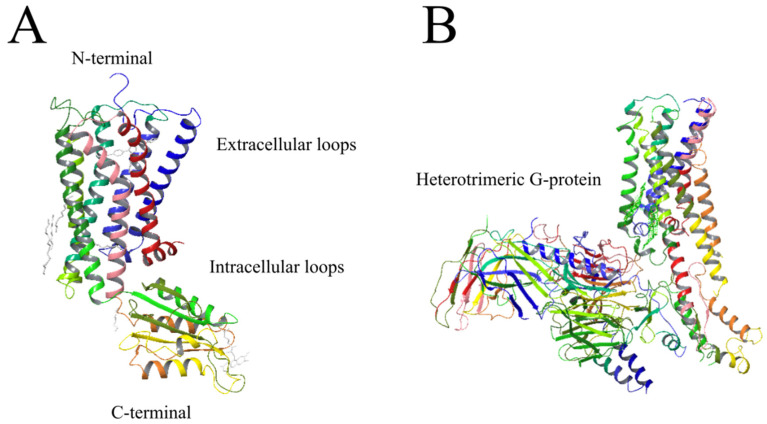
(**A**) Helical structure of CB1-R [85]. (**B**) Helical structure of the G(*i*)-signaling complex made up of the CB1-R and the G(*i*) subunit alpha-1, subunit beta-1, and subunit gamma-2 heterotrimeric proteins [26]. Seven hydrophobic transmembrane segments are connected by loops that extend into the extracellular and intracellular domains alternatively [26]. Modified using Schrödinger Release Software 2021-4: Maestro, Schrödinger, LLC, New York, NY, 2021.

Endocannabinoid receptors that were initially cloned are now well-established target sites and the identity of the cDNA sequence was confirmed when a rat cerebral cortex library revealed the identity of the CB1-R [83]. Studies that focus on CB receptors are critical since the manner in which a ligand interacts with these receptors and signals are transmitted may guide the design of novel drug therapies with more specific cannabinoid ligands [84]. The construction and comparison of three-dimensional helix bundles of mammalian CB receptors depicted in Figure 1 was reported in 1995 [85,86]. CB receptors are heptahelical receptors that couple to guanine-nucleotide-binding proteins and thread through cell membranes at seven sites [87]. The CB1-R and CB2-R receptors are class A G protein-coupled receptors with standard features including a glycosylated extracellular amino-terminal (or N-term) and an intracellular carboxyl-terminal (or C-term). The terminal domains are connected by seven transmembrane domains, precisely three extracellular loops and three intracellular loops [34].

**Figure 2:**
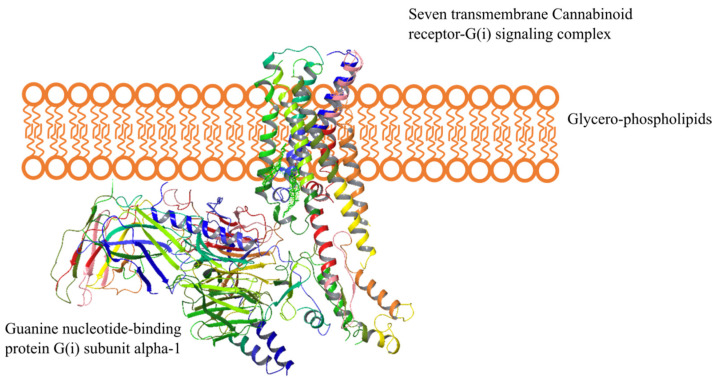
Helical representation of the CB1 receptor. The seven-transmembrane receptor is embedded into the cell membrane, having extracellular helices extending into the extracellular environment and the intracellular helices protruding into the intracellular environment where it interacts with the Guanine nucleotide-binding protein G(*i*) subunit alpha-1. The extracellular domain has an N-terminal that possesses glycosylation sites, while the intracellular domain has a C-terminal coupled to a G-protein complex. Adapted from reference [26]. Modified using Schrödinger Release Software 2021-4: Maestro, Schrödinger, LLC, New York, NY, 2021.

The CB1-R integral membrane protein with seven membrane-spanning helices coupled to heterotrimeric G-proteins on the intracellular side is depicted in Figure 2. The cell membrane is comprised of glycerophospholipids, molecules composed of glycerol, a phosphate group, and two fatty acid chains.

In addition to CB1-R and CB2-R, receptors and ion channels may also interact with the ECS and GPCRs such as GPR55, GPR18, GPR119, peroxisome proliferator-activated receptors (PPAR), transient receptor potential (TRP) channels such as the vanilloid receptor TRPV1, GABA receptors, calcium, potassium, and sodium channels. While the GPCR regulate diverse physiological responses, they share common signaling mechanisms. Extracellular stimuli induce conformational changes in GPCR that activate heterotrimeric G-proteins and other intracellular effectors [88]. The conformational plasticity of some GPCR has revealed the significance of plasticity in GPCR function [89]. Two agonist-bound crystal structures of human CB1-R revealed plasticity in the orthosteric binding pocket that enabled CB1-R to respond to different size and shape of ligands [90].

**Figure 3:**
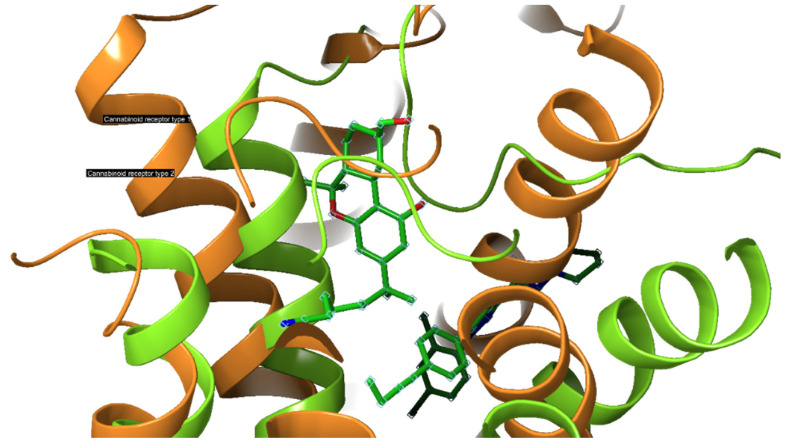
Comparison of the binding pocket of CB1-R and CB2-R. Modified using Schrödinger Release software 2021-4: Maestro, Schrödinger, LLC, New York, NY, 2021.

When the cryogenic electron microscopy structures of the active CB1-R and CB2-R in complex with G*_i_* are compared, the active CB1-R-G*_i_* and CB2-R-G*_i_* are similar and the agonist-binding pockets are almost identical (Figure 3), [2,26,91]. An in-depth review of the differences may illuminate opportunities to develop highly selective ligands that target specific cannabinoid receptors [92]. The structures differ in the direction of movement of transmembrane region 5 (TM5) during activation which is inwards for CB1-R and outwards for CB2-R. The cytoplasmic ends of TM5 and TM6 also differ in the manner of movement following activation, with CB2-R shifting upward relative to CB1-R. The toggle switch residues also exhibit differences for both receptors, with CB1-R possessing a twin toggle switch viz., F200 and W356, whereas CB2-R a single toggle switch viz., W258 that triggers activation and downstream signaling. This suggests that while CB2-R do not undergo change in their conformation following activation, CB1-R exhibit more extensive conformational changes when modulated by agonists. The high plasticity of CB1-R during transition enhances its ability to respond to a variety of ligands when compared to CB2-R [91].

A study conducted using molecular mechanics/generalized born surface area (MM-GBSA) to investigate binding potency and identify hotspot residues for CB1-R and CB2-R revealed that the hotspot residues for CB1-R and CB2-R are similar in both the active and inactive states whereas the hotspot residues of the different active states are diverse [92] suggesting that agonists and antagonists adopt different patterns of interaction. While the key residues contributing to the interaction between agonist and receptor are located in the transmembrane loop 2 (TM2), TM3, and TM5, critical residues of antagonist-bound systems are located in the N-terminal, TM1, TM3, TM5, and TM7.

**Figure 4:**
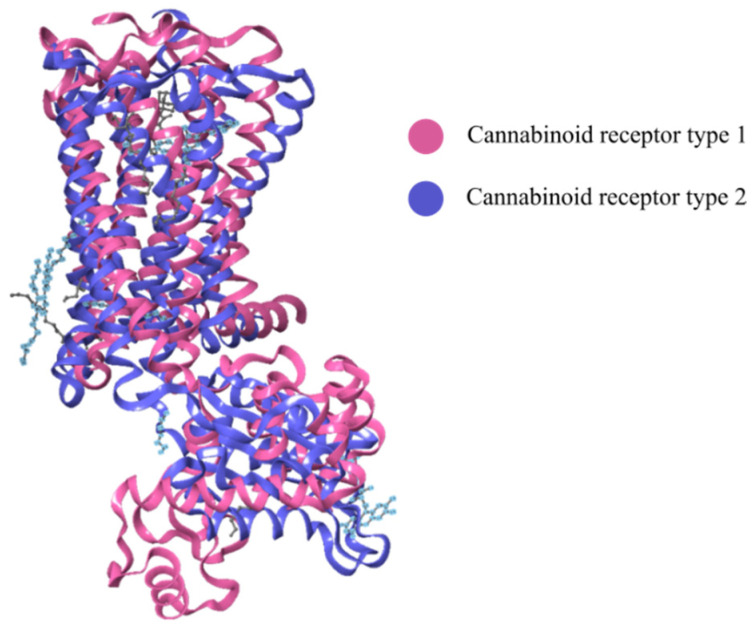
Superposition of AM6538@CB1-R (5TGZ from PDB) (pink) and AM10257@CB2-R (5ZTY from PDB) (purple). The crystal structure of human CB2-R in complex with antagonist AM10257 and crystal structure of human CB1-R in complex with AM6538 shows the conformational lock holding the binding site in the ligand-binding conformation [32,85]. Modified using Schrödinger Release software 2021-4: Maestro, Schrödinger, LLC, New York, NY, 2021 [32,85].

Recent studies have revealed a highly conserved DRY(X)5PL motif, in which the residue Leu-222 resides and is located in intracellular loop 2 of CB1-R and CB2-R, and plays a vital role in mediating selective coupling to G*_s_* and G*_i_* proteins [93,94]. Figure 4 shows a comparison of the gross morphology of the receptors CB1-R and CB2-R. The N-terminal helix of the CB2-R interacting with antagonist AM10257 sits over the orthosteric pocket and does not have direct involvement in antagonist binding. The CB1-R and CB2-R are similar in the conformational lock holding the binding site in the ligand-binding conformation stabilized by an internal disulphide bond in ECL2 [95]. Mutations of the Leu-222 residue in the second intracellular loop of CB1-R, to either Ala or Pro, resulted in a switch in G-protein coupling from G*_s_* to G*_i_*, and when mutated to Ile or Val a switch to a balanced coupling with G*_s_* and G*_i_*.

#### 2.3.1. CB1-R

The expression of CB receptors in the human and rodent brains diminishes according to the natural ageing process [96] and the increased density of CB receptors in the hippocampus is correlated with the disruptive effects on memory and cognition of cannabinoids [97]. The hippocampus is associated with memory and learning processes and chronic Δ9-THC exposure has a negative effect and alters the structure and function of the hippocampus [98]. CB receptors on the basal ganglia play a role in fine-tuning motor control [99] and CB receptor expression and binding decreases in neurodegenerative diseases such as Parkinson’s and Huntington’s disease [100]. Stimulation of CB receptors in the hypothalamus results in an interaction with neuropeptides that regulate energy homeostasis, food intake and lipogenesis in visceral tissues [101]. While CB receptors in the accumbens nucleus stimulate the dopamine reward pathway, CB receptors in presynaptic glutamatergic and GABAergic neurons activate the reward pathway in the ventral tegmental area through retrograde signaling [102]. CB receptors expressed in the dorsal horn of the spinal cord and dorsal root ganglia neurons are involved in pain modulation [103].

CB1 receptors are most abundant in the brain and primarily located in the neurons [104]. CB1-R are expressed with the highest levels of expression in the cerebral cortex, hippocampus, basal ganglia, and cerebellum which corresponds with cannabinoid effects that play a role in cognition and memory locomotion and pain transmission [105]. CB1 receptors are also located in the peripheral tissues including those of the cardiovascular [106], reproductive and gastrointestinal tract systems [107]. The ataxia and immobility experienced after administration of cannabinoids is due to the concentration of CB1 receptors in the cerebellum [108]. The site and density of CB1 receptors are summarized in Table 1. In a study in which FAAH and CB1-R knockout mice were used revealed the primary target that mediates the behavioral effects of anandamide is the CB1 receptor. The human gene locus for the human CB1-R has been identified in chromosome 6 positions 6q14-q15 [109] and encoded by the gene *CNR1* [8]. CB1-R plays an essential role in several pathways, including pain transmission, appetite control, memory, and interaction with the dopaminergic reward center of the brain [84]. Variations of the gene *CNR1* have been associated with cannabis dependence [110,111,112]. Recently the CB1-R was isolated in the mitochondria of striated and heart muscles, where they are directly involved in the regulation of intramitochondrial signaling and respiration [113].

#### 2.3.2. CB2-R

The gene encoding the human CB2 receptor was cloned in 1993 and the location on chromosome 1p36 identified [119]. The site and density of CB2 receptors are summarized in Table 2 and the receptor is abundantly expressed in peripheral tissues of the immune, central nervous, gastrointestinal tract, reproductive and cardiovascular systems, in addition to the liver and bone [120,121,122].

### 2.4. Endocannabinoid Signaling Pathways

**Figure 5:**
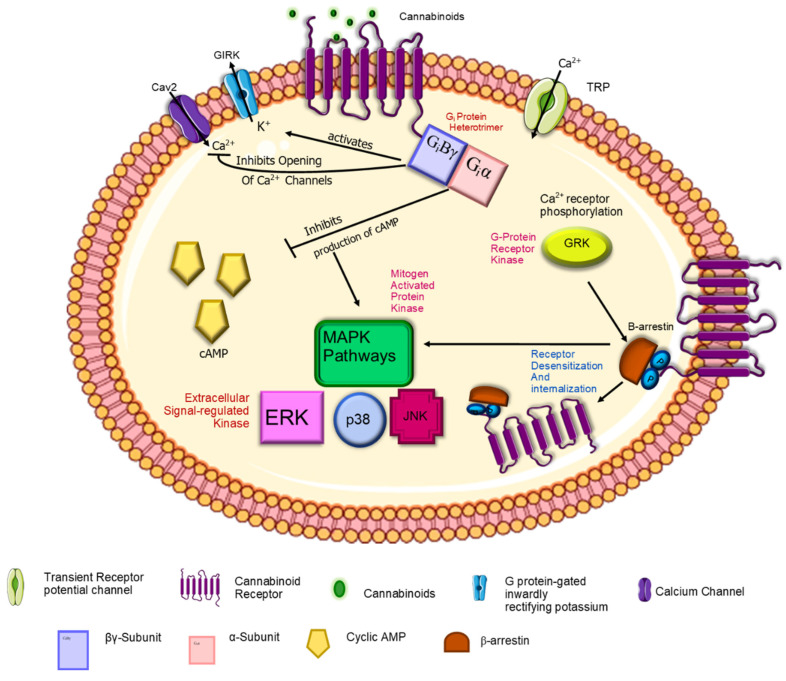
The signal transduction pathways associated with the G*_(i)_* signaling complex. The G*_(i)_* protein comprises three components, namely alpha, beta and gamma subunits. Therefore, they are called the G*_i_* protein heterotrimer. G*_iα_* inhibits the production of Camp G*_iβγ_* activates K+ channels and inhibits the opening of Ca2+ channels. The downstream effects involve the Mitogen-activated protein kinases pathways; ERK, p38 and JNK. β-arrestin attachment to the G*_(i)_* complex initiates receptor desensitization and internalization. Adapted with text and annotation from reference [134] licensed under a Creative Commons Attribution 4.0 (CC BY) license.

Endocannabinoids play an important role as powerful regulators of synaptic function in the central nervous system where they regulate neural functions and behaviors [126]. As fundamental modulators of synaptic function, endocannabinoids can regulate neural functions such as cognition, motor control, pain, and feeding behavior [77]. The interaction between endogenous or exogenous ligands and endocannabinoid receptors initiates signaling pathways. Both CB1-R and CB2-R regulate the action of cAMP pathways and MAPK mechanisms [127]. Figure 5 depicts the main signaling pathways both cannabinoid receptors regulate. Endocannabinoids are referred to as retrograde messengers because the principal signaling mechanism in the endocannabinoid system is retrograde signaling. It has been shown that the movement of endocannabinoids occurs backwards across the synapse after production in the postsynaptic neuron and binding to presynaptic CB1 receptor, which signals suppression of neurotransmitter release [128]. Evidence suggests endocannabinoid signaling also occurs through non-retrograde TRPV1 and postsynaptic CB1 receptors and/or an astrocytic manner [77]. The neurotransmitters controlled by CB1 receptors are the classic neurotransmitters glutamate, GABA and glycine, and neuromodulators including acetylcholine, norepinephrine, dopamine, serotonin, and cholecystokinin [129]. Endocannabinoids also activate the transient receptor potential vanilloid one receptor (TRPV1), which results in the release of a calcitonin-gene-related peptide from perivascular sensory fibers and a vasodilator response in isolated arteries.

#### 2.4.1. CB1-R

Stimulation of the CB1 receptor transduces stimulation of mitogen active phosphorylase kinase (MAP) and adenylyl cyclase inhibition, which decreases cyclic AMP production. CB1 receptors are coupled to ion channels through G*_i_*_/*o*_ proteins [130]. They couple positively to inwardly rectify potassium channels and negatively to N-type and P/Q-type calcium channels. An enhanced outward potassium current is created when cAMP-dependent protein kinases are inhibited due to CB1-R activation. The inhibition of calcium channels is believed to decrease neurotransmitter release from CB1-R-expressing presynaptic terminals [107].

#### 2.4.2. CB2-R

The CB2 receptor inhibits adenylyl cyclase in human lymphocytes and spleen cells in the mouse that express CB2 receptors [104]. The action of CB2 receptors in the immune system is to modulate cytokine release. Stimulation of CB2 receptors present on B- and T-cells lead to reduced responses when the immune system is challenged [131].

G-coupled proteins are secondary messengers that convey information to one or more effector proteins following receptor binding [132] and modulate the release of $Ca^{2+}$ from intracellular stores which then bind to and regulate ion channels. The heterotrimeric G-protein can be coupled to three subunits viz., α, β and γ, which act independently in downstream signaling systems. When an agonist stimulates the receptor, a conformational change of the receptor allows for interaction with intracellular G-proteins which results in dissociation of the subunits from the G-protein. The dissociated dimer subunit G*_i_α* proteins regulate adenylyl cyclase. The inhibition of cyclic AMP production was noted when cell lines expressing CB1 or CB2 receptors were stimulated [133]. The free dimer β_γ_ mediates the regulation of ion channels, mitogen-activated protein kinase (MAPK) and phosphatidylinositol-3-kinase (PI3K). The two most studied endocannabinoid receptors CB1-R and CB2-R are separated primarily by differences in their amino acid sequence signaling mechanisms and tissue distribution.

### 2.5. Synthesis and Degradation of Endocannabinoids

The first two endocannabinoids to be discovered were N-arachidonoylethanolamine (anandamide) and 2-arachidonoylglycerol (2-AG) which are both synthesized on demand in response to elevated intracellular calcium levels [135]. While evidence has emerged for the existence of at least 15 additional endogenous cannabinoid compounds [136], in this review we will only discuss the biosynthetic and catabolic pathways of the two most studied endocannabinoids, anandamide and 2-AG. Anandamide, a retrograde messenger, synthesis occurs in the postsynaptic neuron. The biosynthetic precursor of anandamide is N-arachidonoyl-phosphatidylethanolamine (NArPE), and the most likely precursor of 2-AG are the diacylglycerols, DAG-_α_ and DAG-_β_ with an arachidonic acid moiety at position 2 [76]. The precursor NArPE is produced from N-arachidoylation of phosphatidylethanolamine due to the transfer of arachidonic acid from the sn-1 position of phospholipids to the nitrogen atom of phosphatidylethanolamine. The precursors DAG-_α_ and DAG-_β_ are produced from either the phospholipase-C-catalyzed hydrolysis of phosphatidylethanolamine or from the hydrolysis of phosphatidic acid [137]. Anandamide is metabolized by FAAH through hydrolysis of the amide bonds and 2-AG degrades by multiple monoacylglycerol lipases activity which hydrolyses ester bonds [138]. Several enzymes rapidly degrade the endocannabinoid 2-AG including cytochrome P450, cyclooxygenase-2, lipoxygenase, and α/β hydrolase domain-containing proteins 6 and 12 (ABHD6/12) [139,140].

### 2.6. Cannabinoids

A 2008 review described cannabinoids as the terpenophenolic constituents of the hemp plant [84]. The cannabinoids are classified broadly into four categories loosely based on their origin viz., phytocannabinoids, endocannabinoids, synthetic cannabinoids and cannabimimetics. The chemical structure and receptor affinity of some common phytocannabinoid, endogenous cannabinoids and synthetic cannabinoids are depicted in Table 3. While classic cannabinoids are found in the plant extracts of *Cannabis sativa*, they are also naturally produced in the mammalian body as endocannabinoids in a characteristic on-demand biosynthetic pathway. Endocannabinoids are similar to prostanoids in that they are not stored but are rather generated upon demand in response to a depolarization-induced rise in intracellular calcium levels or following activation of different metabotropic receptors [141]. Phytocannabinoids are plant-derived products that stimulate cannabinoid receptors by directly interacting with G*_i_*/G_o_-coupled protein cannabinoid receptors or by exhibiting structural similarities with cannabinoids [142]. *Cannabis sativa* L. is a dioecious plant belonging to the Cannabaceae family. Cannabinoids, flavones, and terpenes are the main phytochemicals found in this plant. Due to synergistic activity some biological activities of cannabinoids are known to be enhanced due to the presence of terpenes and flavonoids in the extracts [143]. Phytocannabinoids are natural terpenoids or phenolic compounds derived from *Cannabis sativa* such as alkaloids, terpenes, terpenoids, polyphenols and derivatives of fatty acids, present in plants not belonging to the cannabis genus. The most potent psychoactive constituent of *Cannabis sativa* is Δ9-THC, first isolated in 1964 [144]. The Δ9-THC is rapidly absorbed and converted in the lungs and liver to the active metabolite, 11-hydroxy-Δ9-THC. Another principal constituent of the cannabis plant is cannabidiol (CBD), which was successfully isolated in 1940 [145].

As research into the ECS demonstrated cannabinoid receptors as viable targeting sites, more and more natural products are emerging as potential therapeutic prospects. Novel cannabinoids from diverse natural sources and species, including animal venom which contain toxins that modulate CB1-R and CB2-R with high affinity and selectivity [2]. The novel cannabinoids from animal sources are referred to as peptide-type cannabinoids, which are part of the hemopressin peptide family and are derived from the α and β chains of haemoglobin [146].

The components of the endocannabinoid system, including cannabinoids and related lipid mediators, make up the endocannabinoidome we have come to know today. There is an ever-growing list of cannabinoids, including phytocannabinoids from the cannabis plant, endogenous compounds, synthetic analogues, phytogenic and synthetic cannabimimetics that have been studied in recent years [147]. Phytocannabinoid dietary intake of β-Caryophyllene, Quercetin, Resveratrol, Kaempferol and Biochanin A, is being studied as a viable strategy for disease prevention [148,149].

**Table 3:** Chemical structures of cannabinoids and their affinity to the CB receptors.

Cannabinoid	Receptor Affinity	Reference
Phytocannabinoids		
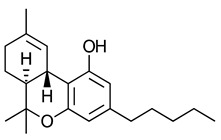 $\Delta^{9}$-$\mathrm{tetrahydrocannabinol}\;(\Delta^{9}$-THC)	CB1-R and CB2-R agonist	[150]
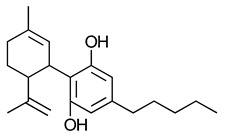 (-)-Cannabidiol (CBD)	No activity at CB1-R or CB2-R	[150]
Endogenous cannabinoids		
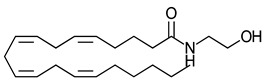 Anandamide	Greater CB1-R than CB2-R agonist TRPV_1_ agonist	[104]
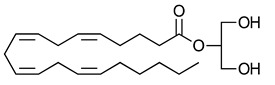 2-Arachidonoyl glycerol	CB1-R and CB2-R agonist	[70]
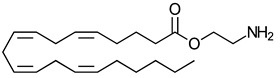 Virodhamine	Greater CB1-R than CB2-R agonist	[150]
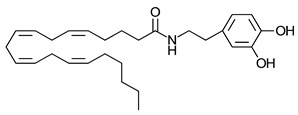 N-Arachidonoyl dopamine	Greater CB1-R than CB2-R agonist TRPV_1_ agonist	[150]
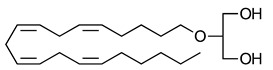 Noladin-ether	Greater CB1-R than CB2-R agonist	[150]
Synthetic cannabinoids		
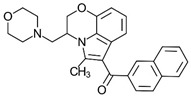 Aminoalkylindole WIN 55,212-2	Highly selective CB2-R agonist.	[151]
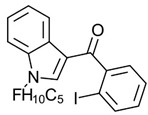 Benzoylindole AM694	Potent CB receptor agonist. Highly selective for CB2 receptor.	[152]
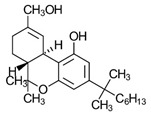 Dibenzopyran HU-210	Highly potent CB1-R and CB2-R agonist. Preference for CB1-R receptors.	[153]
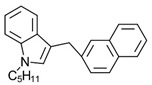 Naphthylmethylindole JWH-175	Selective CB1-R agonist.	[154]
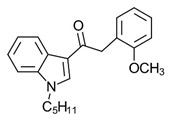 Phenylacetylindole JWH-250	Potent CB agonist with greater affinity towards CB1-R.	[155]

### 2.7. Selection of Cannabinoid Ligands for Specific Targeting

Cannabinoid receptors are important for regulating a variety of physiological and pathological activities and potential ligands for the receptors hold promise as drug candidates for the treatment of different diseases. Ligands have been developed with functional and subtype receptor selectivity with the help of structural studies of cannabinoid receptors that provided insight into ligand-receptor interaction, activation and/or signaling of cannabinoid receptors. Structural information relating to cannabinoid receptors has permitted researchers to design specific ligands for the precise modulation of the endocannabinoid system [156]. A study on the structure-activity relationships of several classes of cannabinoid ligands demonstrated that ligands possessed high affinity with good biological efficacy were possible via the lipophilic regions necessary for activity [157]. When examining the effects of amide substituents with pyrazole rings on receptor affinity pyrazoles were found to be CB1-R selective.

The most commonly studied cannabinoid ligands activate or inhibit cannabinoid receptor signalling by binding at the orthosteric sites of cannabinoid receptors [158]. The orthosteric sites are active sites located on endogenous cannabinoid receptors to which endogenous cannabinoid receptors bind. The challenge with orthosteric ligands is associated adverse effects which including psychotropic and psychiatric effects for CBR-1 agonists, CB1-R antagonists, and inverse agonists [159].

Allosteric modulators bind to spatially distinct binding sites at the CB1 and CB2 receptors [160]. Allosteric modulators may be grouped into positive allosteric modulators (PAM) which enhance the effect of endogenous cannabinoids or co-administered orthosteric cannabinoids and negative allosteric modulators (NAM), which diminish the response [161]. The ability to fine-tune physiological responses of cannabinoid receptors in the presence of endogenous ligands is a feature of PAM or NAM activity. Another characteristic of the compounds is the increased receptor subtype specificity [162].

#### 2.7.1. Allosteric Modulators

Allosteric modulators do not activate the receptor directly but interact with topographically specific sites from the orthosteric ligand-binding pocket which modify the structural conformation of the respective receptor protein [163]. Modern technologies in receptor studies have facilitated a better understanding of the manner by which allosteric modulators modulate the affinity and efficacy of orthosteric ligands [164]. Bitopic ligands are molecules that interact with both orthosteric and allosteric sites and thereby engender selectivity through concomitant engagement with both sites on a single GPCR. The bitopic ligand is a single chemical entity with an orthosteric and allosteric pharmacophore chemically attached via a linker [165].

#### 2.7.2. Positive Allosteric Modulators (PAM)

An approach to improving the safety profile of direct orthosteric activation is the pharmacological modification of receptor activation using PAM to enhance the activity while maintaining spatial and temporal control of the receptor. The enhanced signalling action of PAM may be through an increase in receptor-orthosteric ligand affinity or less efficient dissociation of orthosteric ligand and receptor [166].

Pamplona et al. [167] reported lipoxin A4, an endogenous anti-inflammatory ligand, enhances the activation of CB1 receptors when induced by AEA. The allosteric enhancement by lipoxin A4 occurs when administered exogenously or produced endogenously.

A study investigating the effects of positive allosteric ligands of cannabinoid receptors using a potent dual orthosteric agonist revealed that when used to treat neurodegenerative disorders, a combination of the orthosteric agonist and positive allosteric modulator was a promising therapeutic approach [168]. GAT229, the positive allosteric modulator, has been reported to modulate CB1-R and EC21a has been reported to modulate CB2-R through the allosteric site of the CB2 receptor. GAT229 is known as a pure CB1-R PAM and is the S enantiomer of the 2-phenylindole derivative GAT211 [169]. EC21a is the first synthetic CB2-R PAM, EC21a established to enhance the ability of CP55940 and 2-AG but not that of AEA [170]. EC21a also stimulated the [35S] GTP (gamma)S subunit which binds to CB2-R. The authors reported EC21a strongly potentiated the effect of the CB2-R agonist in an in vivo model and did not induce any effect in the absence of the CB2-R agonist. Swiss albino mice, inbred C57BL/6 mice, CB1-R knockouts and controls, and 5-LOX knockouts were used in the experiments. In addition, an enhanced effect of CB1-R activation when coupled with PAM GAT229 and the cannabinoid agonist was observed.

#### 2.7.3. Negative Allosteric Modulators (NAM)

NAM have been shown to weaken the impact of orthosteric ligands and Shao et al. [171] revealed the CB1-R response to allosteric modulators in respect of cannabinoid binding and efficacy. Further the crystal structure of CB1-R with the negative allosteric modulator ORG27569 and the agonist CP55940 were successfully solved. The structural data revealed that NAM binds to an extrahelical site within the inner leaflet of the membrane, making the agonist-binding pocket adopt an inactive conformation, despite significant contraction of the pocket.

#### 2.7.4. Functional Selectivity

Biased agonists produce different degrees of activation along different pathways. Following activation, a biased agonist can selectively activate either the G protein or β-arrestin pathway [172]. Cannabinoid receptor biased agonists such as PNR-4-20 stimulate G-protein mediated signaling but is less efficient in respect of β-arrestin two recruitment [173]. β-arrestin two recruitment is associated with the internalization of CB1-R [174]. LY2828360 is another ligand that produces biased signaling, when compared to CP55,940 also produced a lower degree of CB2 receptor internalization [175].

#### 2.7.5. Peripherally Acting Cannabinoids

An attempt to bypass the side effect profile of direct CB1-R activation led to the development and clinical investigation of the peripherally restricted CB1-R and CB2-R agonist, AZD1940 [176]. AZD1940 is a novel synthetic CB1-R and CB2-R agonist with high binding affinity and a full agonist of human, rat and mouse CB1-R and CB2-R receptors [177].

The activation of peripheral CB1-R has been proposed to exhibit analgesic activity for which the analgesic properties of AZ11713908, a peripherally restricted CB1-R agonist was evaluated following by systemic administration of AZ11713908 in CB1-R and CB2-R knock-out mice [178] which produced robust analgesia via peripheral CB1-R in rodent pain models.

## 3. Toolkit of Therapeutics of ECS

**Figure 6:**
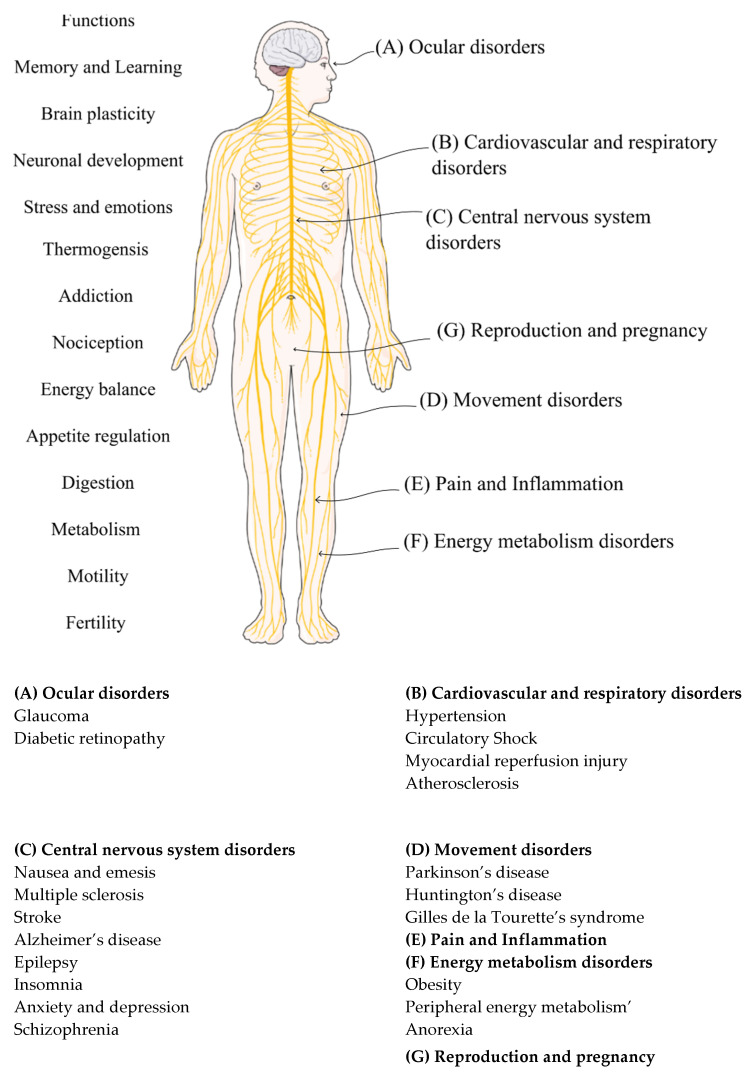
The functions and pharmacotherapy targets of the endocannabinoid system [150]. Figure modified with text, and annotation after adaptation of “Nervous System” from Servier Medical Art by Servier, licensed under a Creative Commons Attribution 3.0 Unported License [180] and reference [181] with permission.

The complexity of cannabinoids demands a deep understanding of molecular pharmacology to facilitate fine-tuning of potential cannabinoid treatments. Understanding the endocannabinoid system also permits identification of new potential leads for drug development [172]. An arsenal of therapeutic tools is already available viz., (I) inhibitors of cellular uptake of FAAH and MAGL endocannabinoid; (II) selective CB1-R and CB2-R agonists and antagonists; (III) anandamide analogues and (IV) dual CB1-R/CB2-R agonists. Experimental models of different disorders involving the endocannabinoid system revealed the ECS to exhibit a protective function or a contribution to the symptoms and progression of disease [179]. Several possible functions of the endocannabinoid signaling system in respect of physiological and pathological conditions are highlighted in Figure 6.

By regulating two elementary signaling molecules viz., neurotransmitters from neurons and cytokines from immune cells, the endocannabinoid system is believed to play a pro-homeostatic function in pathology [43,182,183]. The biological effects of endocannabinoids intricately associated with cannabinoid and non-cannabinoid receptors in addition to crosstalk involving the arachidonic acid metabolic network and epigenetic regulation of homeostatic synaptic systems [184,185]. Changes in the endocannabinoid system have been observed for different pathophysiological conditions due to changes in cannabinoid receptor density and coupling efficiency [186]. Pathophysiological conditions have also been noted when endocannabinoid levels are altered through synthetic and endogenous endocannabinoid enzyme degradation activity [187]. The pro-homeostatic role of the endocannabinoid system is supported by the anatomical distribution of the protein in different organs and tissues. Regarded as a pleiotropic but locally acting signal system, the ECS is activated on-demand following disturbance of the homeostasis of the local environment to re-establish balance [76]. Under some pathological conditions, the ECS can become dysregulated and subsequently contributes to the progression and/or symptoms of disease [182].

The anti-proliferative properties of cannabinoids in which inhibition of adenocarcinoma cell growth after oral administration of Δ9-THC in mice has been reported [188]. The endocannabinoid signaling system controls neoplastic cells, modulates proteins and the nuclear factors involved in cell proliferation, differentiation, and apoptosis [189]. The neoplastic activity of cannabinoids affects signaling pathways that are essential for cell growth, survival and inhibition of angiogenesis or direct initiation of apoptosis or cell arrest [190]. The upregulation of CB2 receptors has been reported in Alzheimer’s, Huntington’s, encephalitis, and multiple sclerosis [191]. A reduction in CB1 receptor expression or mRNA in glial tumors results from neuronal loss, similar to the reduction observed in neurodegenerative diseases and the normal ageing process [150].

While enhanced endocannabinoid tone may result in cessation of disease, there are cases in which up-regulation of the system is involved in pathogenesis [43] of obesity when stimulation of the CB1 receptor results in an increase in food intake, both in systemic administration and when anandamide is administered into the ventromedial hypothalamus [192]. The administration of 2-AG into the shell of the nucleus accumbens or lateral hypothalamus exhibited a similar increase in food intake. The sites of orexigenic action of endocannabinoids in both the hypothalamus and the limbic forebrain are correlated with brain involvement in both the homeostatic and hedonic control of eating [193].

The ECS is known to impact the female reproductive system where it has an effect on oocyte maturation, folliculogenesis, and ovarian endocrine secretion [194]. A complex interplay between the hypothalamic-pituitary-ovarian axis and ECS, as well as an intricate crosstalk between the ECS and steroid hormone production contributes to oviductal embryo transport, uterine decidualization, implantation, and placentation in the female reproductive system [195,196]. Endocannabinoids have been found to regulate male reproduction by acting at central and gonadal levels, they modulate the pituitary-gonad axis [197,198]. The cannabinoid THC has been found to modulate neurosteroidogenesis following direct activation of the CB1-R, by stimulating the synthesis of pregnenolone from cholesterol through negative feedback mechanism. This activation of the CB1-R results in increased pregnenolone levels in the brain [199].

### 3.1. Enhanced Endocannabinoid Tone

While some cannabinoid receptor ligands such as allosteric modulating agents and biased agonists act directly), other treatment options indirectly target the enzymes responsible for synthesis and catabolism [172]. In many pathophysiological conditions such as inflammation, neurodegeneration, gastrointestinal tract irritation, metabolic and cardiovascular diseases, pain, and cancer the endocannabinoid system is upregulated to prevent progression of disease by serving an auto protective role [200]. To take advantage of this autoprotective function, enhancing endogenous endocannabinoid tone through inhibiting endocannabinoid degradation, uptake, and intracellular transport is a strategic approach for the treatment of many diseases [43].

Pharmacological strategies for enhancing endocannabinoid tone include additional targets outside the endocannabinoid system, specifically cyclooxygenase-2 (COX), cholinesterase (ChE), prostaglandin F2α receptor (PGF2α-EA) in addition to commercially available medicines and plant material which exhibit cannabimimetic properties that enhance endocannabinoid levels [201]. Standard structural features of endocannabinoids and other lipid mediators such as prostaglandins and leukotrienes are also formed from arachidonic acid and share some of the metabolizing enzymes of endocannabinoids. These materials are considered endocannabinoid-like compounds or cannabimimetics and can modulate cannabinoid signaling through structure-related lipid messengers. The potentiating action of endocannabinoids is sometimes termed the entourage effect [202,203].

#### 3.1.1. The Inhibition of Endocannabinoid Degrading Enzymes

The inhibition of endocannabinoid metabolism focusses on two enzymes viz., fatty acid amide hydrolase (FAAH) and monoacylglycerol lipase (MAGL) [187]. The main endocannabinoids of interest, anandamide and 2-arachidonoyl glycerol, can also be metabolized by N-acylethanolamine hydrolytic acid amidase and serine hydrolases (MAG kinase, α, β-hydrolase (ABHD6 and ABHD12) respectively. In addition, both the endocannabinoids are suitable substrates for cytochrome P450 lipoxygenases and cyclooxygenase-2 enzymes [204]. FAAH inhibitors as potential therapeutic agents have been described recently [205]. The ability to elevate local endocannabinoids under certain stimuli and minimize the unwanted secondary effects of global activation of cannabinoid receptors has been an area of interest for researchers, but none have been commercially successful, to date. The serine hydrolase monoacylglycerol lipase is upregulated in primary tumors and cancer cells. MAGL inhibitors have been found to impair cell migration, tumorigenicity and invasiveness, and therefore are important in many therapeutic fields [206].

#### 3.1.2. The Inhibition of Endocannabinoid Uptake and Intracellular Transport

A strategic approach to potentiating endocannabinoid signaling is the prevention of endocannabinoid inactivation by intracellular enzymes through inhibition of extracellular uptake of endocannabinoids. Compounds which inhibit the cellular uptake of anandamide include AM404, VDM11, UCM707, OMDM1, OMDM2, and LY2183240 [42]. Retrograde endocannabinoid signaling causing the release of endocannabinoids into the synaptic cleft immediately after synthesis. The nature of retrograde signaling not only requires the efficient transport of endocannabinoids across the lipid bilayer away from their biosynthetic enzymes, but also translocation across the synaptic cleft in order to activate presynaptic CB receptors and control synaptic transmission and plasticity [207].

Several endocannabinoid membrane transporter (EMT) inhibitors have been tested in preclinical studies. While most EMT inhibitors were thought to be specific for EMT, they have been shown to interact with other components of the ECS and inhibit FAAH, MAGL, COX-1, 2, and FABP5. Some highly potent EMT inhibitors with good selectivity include Guineensine, BSL34, WOBE437, and RX055. Guineensine is extracted from the medicinal plant *Piper nigrum* and demonstrated cannabimimetic effects in mice, proving it is a highly potent inhibitor of anandamide and 2-AG. Naturally occurring fatty acid amides in *Echinacea puprurea* exhibited anti-inflammatory, analgesic, and anxiolytic properties in mice and WOBE437 and RX055 are synthetic derivatives of these fatty acid amides [208]. There are two prevailing models in which endocannabinoids are transported across the membrane, either simple diffusion or facilitated diffusion by a putative transmembrane transporter [207].

Another approach to enhance endocannabinoid tone, is to block the intracellular proteins involved in endocannabinoid transport. ARN272 competes with AEA for FLAT binding, thereby reducing the internalization of AEA [209] and SBF126 inhibits the uptake of AEA by binding to the fatty acid-binding proteins FABP5 and FABP7. The phytocannabinoids Δ9-THC and CBD can also enhance endocannabinoid levels by inhibiting degradation through competition for binding to intracellular carriers of FABP [210].

It has been shown that the saturable, temperature-dependent substrate selective properties of lipophilic endocannabinoids make the uptake of endocannabinoids possible through facilitation by proteins [201,210]. Cellular studies suggest that endocannabinoids, Anandamide, and 2-AG are substrates for intracellular binding and transport proteins. Lipid binding proteins including members from the fatty acid binding protein (FABP) family, albumin, and heat shock protein 70 have been characterized as endocannabinoid transport proteins that bind, sequester, and traffic endocannabinoids contributing to their availability to activate cannabinoid receptors [211].

### 3.2. Sites and Tissue Specificity of Endocannabinoid Receptor Distribution

While cannabinoid receptors are distributed widely throughout the body of vertebrates, there are differences in anatomical distribution [187]. In most pathophysiological cases, the ECS is up-regulated however, the levels of endocannabinoids and expression of CB-R may present differently depending on the tissue or experimental model used [43,183]. The impairment of the ECS has been linked to pathological conditions (Section 3) suggesting that modulation of this system may be essential in disease treatment and the maintenance of health status. Evidence has shown the epigenetic modulation of the ECS in biological tissues induce epigenetic changes that affect health maintenance and influence disease load and resistance [212]. The main targets in epigenetic modulation of the ECS in response to environmental factors appear to be *CNR1*, the gene encoding CB1-R and FAAH, the hydrolyzing enzyme with subsequent alteration of endocannabinoid signalling or tone [213,214,215]. Epigenetic changes have also been noted in pathological states including Alzheimer’s disease, colorectal cancer, and glioblastoma [216,217,218]. The use of endocannabinoid receptor synthetic agonists or antagonists, phytocannabinoids, and endocannabinoids have proven to affect epigenetic mechanisms in animal models, humans, and cell lines [219,220,221,222].

The homology of CB1-R between mice, rats, and monkeys is identical to human CB1-R (97–100%) and lower homology occurs for CB2-R with only 81% similarity between the rat and human receptors. The action of different cannabinoids differs with cellular context and species differences [223]. Human CB1-R and mouse CB1-R are pharmacologically different in profile than that of CB1-R in the rat [224]. Significantly different pharmacological profiles are noted between human, mouse, and rat CB2-R [225].

In the same pathophysiological state, it has been shown that the concentration of endocannabinoids differs depending on species, stage of disease and tissue involved [43]. In a tragic mishap following the administration of a FAAH inhibitor during a clinical trial in France, four volunteers suffered permanent brain damage, and one volunteer died [226]. Health agencies, including the United States Food and Drug Administration, European Medicines Agency and the National Agency for the Safety of Medicines and Health Products pooled resources to investigate this tragedy and established that the possible causes were due to off-target effects of the drug [227].

A potential strategy to target the ECS specifically is the use of FABP since they display specificity in tissues at selected sites in the body [208,228]. Cell-penetrating peptide mediated cellular uptake is the mechanism where cell-penetrating peptides (CPPs) penetrate through cell membranes and are subsequently internalized in cytoplasm or nucleolus [229]. Another approach involves the use of allosteric modulators, which are more effective than orthosteric ligands with respect to specificity, target selectivity and saturability [159]. The tissue-specific action of allosteric cannabinoid ligands occurs because allosteric modulation controls the receptor response only in those tissues that contain the endogenous orthosteric ligand [160].

### 3.3. G-Protein Subtype and β-Arrestin Specificity

**Figure 7:**
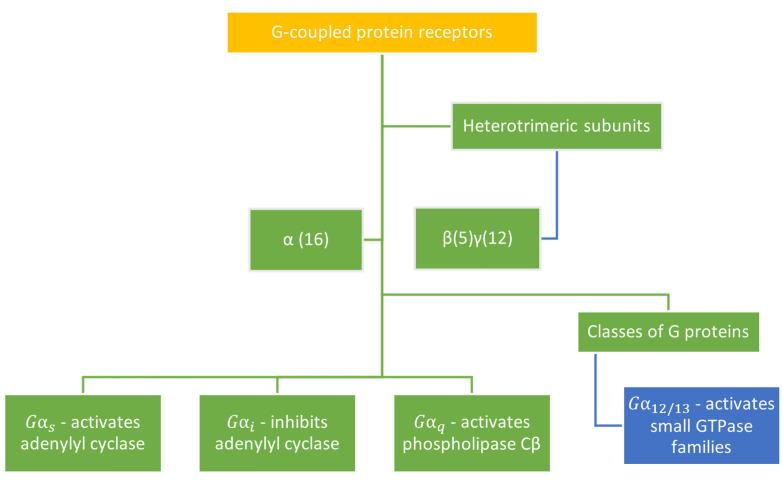
The four families of G-proteins interact differently with effector proteins to initiate signaling [233].

Each guanine protein interacting with cannabinoid receptors is coupled to effector proteins or subunits that mediate cellular signals. The heterotrimeric subunits and classes of G-proteins are depicted in Figure 7. Researchers have only recently begun to quantitate the ligand bias of cannabinoid-targeted compounds. While CB1-R and CB2-R preferentially couple to G*_i_* proteins, CB1-R also couples with G*_s_* or G*_q_* proteins [230]. In a study in which the agonist WIN 55,212-2 was used the quantification of Gα*_i_* and βγ subunit proteins coimmunoprecipitate with the CB1-R revealed that WIN 55,212-2 behaves as an agonist for all three G*_i_* subtypes. In addition, ligand-selective G protein responses were identified, and multiple receptor conformations may result in individual G protein activity [231]. To test the specificity of cannabinoid receptor agonists in activating G*_s_* or G*_i_* –coupled pathways, Bonhaues et al. [232] investigated and quantitated the potency and intrinsic activity of different receptor ligands in stimulating or inhibiting cAMP. Marked differences were observed for the cannabinoid receptor agonists in respect of an ability to activate intracellular transduction pathways and trafficking of receptors. Structural determinants of the cannabinoid receptors revealed that while CB2-R selectively coupled to G*_i_* and inhibited cAMP production, CB1-R coupled to both G*_s_*-mediated cAMP production and G*_i_*-induced activation of ERK1/2 and Ca^2+^ transportation [93].

Ligand-receptor complex activation leads to conformational changes in the receptor which permits binding and activation of heterotrimeric G-proteins. G-protein-coupled receptor kinases recognize the structural conformation of cannabinoid receptors and undergo differential phosphorylation to generate specific patterns or barcodes depending on the ligand [234,235]. The barcode generated are detected by β-arrestins and are recruited to the plasma membrane where G protein-receptor interaction and uncoupling the receptor and G protein are sterically hindered. This process is also called desensitization and is known as the end of the first spatiotemporal wave. β-arrestins initiate the second wave involving internalization of the receptor and the third and final wave occur in the intracellular compartment where the receptor can reengage with the G-protein or β-arrestin [236].

β-arrestins act as scaffold proteins for endocytic machinery and signaling molecules including mitogen-activated protein kinases (MAPK). The ligand-induced receptor internalization role of β-arrestins occurs as the β-arrestin C terminus binds directly to clathrin, thereby producing a scaffold for endocytic machinery. In this way, desensitized receptors are internalized from the cell surface via clathrin- or caveolae- mediated endocytosis.

In a study involving the frog Pelophylax esculentus, the endocannabinoid AEA was found to modulate the GnRH system in vitro and downregulate steroidogenic enzymes in vivo [237]. Another possible regulator of GnRH secretion is the kisspeptin receptor (G-Protein coupled receptor 54) and the ligand kisspeptin. Important events in kisspeptin-dependent GnRH secretion include kisspeptin activated intracellular signaling pathways through the GPR54 [238]. An investigation into the downstream action of GnRH secretion reported that kisspeptin-dependent luteinizing hormone secretion involves β-arrestin-dependent signaling that plays a role in reproductive function via GPR54 [239]. The KiSS-1/GPR54 system coupled to Gαq/11 regulates the hypothalamus by activating phospholipase C hydrolysis. The secondary messengers inositol-1, 4, 5-trisphosphate and diacylglycerol as well as arrestin-1 and arrestin-2 contribute to hormone release [240,241,242,243]. While the ECS modulates the hypothalamic release of GnRH and has consequences on steroid biosynthesis in several species [198], and the expression of CB1-R has been reported in different subpopulations of *kiss1* neurons in female mice [244], currently the possible correlation between the kisspeptin/GnRH system and the ECS has only been demonstrated in the hypothalamus and gonad of male amphibians [237]. Apart from the involvement in the hypothalamus the ECS also permits gametogenesis and extragonadic maturation of the male gamete [198].

### 3.4. Homology Modelling, Molecular Docking and Molecular Dynamics Simulation

The increasing acceptance of phytocannabinoids across the globe has resulted in a need to identify their role in the management of disease. Precise mapping and localization at the cellular, tissue and regional levels of distribution of cannabinoid receptors is critical to determining their role accurately [245]. The challenge with developing drug products with *Cannabis sativa* is finding suitable models to test the therapeutic efficacy since few animals exhibit the same endocannabinoid system biodistribution and activity as humans [245].

Mapping the genome and elucidating the structure of cannabinoid receptors with the aid of modelling software may facilitate an understanding of how ligand-receptor complexes engage in modulating downstream signaling of the ECS. Consequently, the elucidation of cannabinoid receptors and other GPCR structures is an attractive area of research [246]. Breakthroughs in this field include techniques such as microfocus diffraction beamlines, lipidic cubic phase crystallization, and T4 lysozyme insertion. Along with x-ray crystallography, and electron crystallography, electron paramagnetic resonance, ultraviolet absorbance, fluorescence and nuclear magnetic resonance (NMR) spectroscopy have facilitated the resolution of receptors at an atomic level [247] resulting in determination of the 3D structure of GPCR in addition to labelling ligands and selected residues in the binding pocket and elucidate the conformational changes and movement following activation of the receptor [88,248]. To depict the molecular basis for GPCR signaling accurately, an understanding of how a GPCR changes shape over time is essential [89]. Molecular dynamics simulations at an atomic level can be used to predict the motion of every atom in a receptor and those in the molecules with which the receptor interacts [249].

## 4. Improving Drug Delivery through Use of Nanoprecision Tools

The major challenge inhibiting the progression of clinical use of cannabinoids is the challenge of formulation design of pharmaceutical dosage forms to incorporate the cannabinoids which exhibit low aqueous solubility, low bioavailability, are oily and resin in nature which are unstable. The untapped potential of the endocannabinoid system is often restricted by unspecific site targeting. Attempts to develop peripheral or CB2-R selective agonists to minimize centrally occurring psychoactive effects have not yet been successful. Clinical studies revealed a loss in translation between the reality of clinical studies and the preclinical promise of CB2-R agonists in experimental models [208,209]. Nanotechnologies have, however, been successfully developed elsewhere for site-specific targeting [250,251]. Nanoprecision tools, including stimuli-activated nanoparticles, nanobots and other intelligent nanocarriers, have successfully delivered payloads to the targeted site of action [252].

Pharmaceutically, nanotechnology is a popular alternative to traditional approaches of medicine delivery due to an ability to engineer the technology for improved efficacy [253]. Commonly explored nanocarriers include carbon nanotubes, dendrimers, micelles, liposomes, nanoshells and polymeric nanocarriers [254]. Nanoprecision tools may be used for more efficient delivery of highly lipophilic drugs, protection from harsh environments, targeted and controlled delivery to achieve precise biodistribution to a specific site over a predetermined time [255]. Traditional drawbacks of pharmaceutical nanocarriers are instability during circulation, low uptake by target cells and low renal clearance [256]. Another important consideration is the immune response to nanocarriers which results in uptake by immune cells [257]. Cellular uptake has been found to depend on physicochemical properties such as surface modification, size, and shape of the nanotechnologies [258].

The ideal drug delivery vehicle (DDV) platform may be strategically engineered to pass biological barriers and facilitate specific intracellular localization by modulating drug release mechanism, mode of delivery and transport into the body, and target cells [259]. Physical cues govern the interaction between nanoparticles and cells [260]. To avoid the mononuclear phagocyte system (MPS) and ensure a longer circulation time, nanoparticles of <100 nm are more efficient. The geometric shape is also known to affect cellular uptake, in vivo fate and hemorheological dynamics of nanoparticles. The surface charge of nanoparticles also affects the biodistribution and blood circulation time whereas the chemical composition, biocompatibility, size, and surface properties of nanoparticles may impact cellular uptake [261]. It has been demonstrated that a resistance to deformation or particle rigidity also affects cellular uptake and efficacy of treatment [262].

When designing a GPCR-mediated targeted drug delivery system, entry into the cell across biological barriers is an essential consideration [263]. Cell transport mechanisms are active or passive and involve simple or facilitated diffusion, exocytosis, endocytosis, active transport, or osmosis [264]. Endocytosis or cellular internalization is a physiological process by which concerning nutrients enter the cell or the destruction of pathogens is directed. The primary mechanisms include phagocytosis, pinocytosis, and receptor-mediated endocytosis. Phagocytosis is associated with the uptake of solid particles and pinocytosis, the passage of fluid-phase particles through the membrane which are non-selective mechanisms. In contrast receptor-mediated endocytosis is a selective process of internalization. The mechanisms of interest for nanocarrier-based targeted drug delivery design is phagocytosis. The strategies used to bypass lysosomal destruction of drugs and receptor-mediated endocytosis where specific transport into the cells through clathrin, caveolin, or clathrin- and caveolin-independent pathways will be discussed [265].

Receptor-mediated drug delivery systems (DDS) are well-established approaches used to improve specific targeting and enhance cellular uptake of therapeutic compounds [266]. The importance of targeted drug delivery systems difficult to treat diseases is necessary after non-specific oncolytic agents wreaked havoc to non-cancerous cells while treating malignant tumors [267]. Such technologies may increase accumulation of drugs within specific tissues, enhance diagnostic imaging and ensure a prolonged half-life is exhibited in vivo when compared to non-targeting DDS [268]. In an effort to enhance drug accumulation in tumors, researchers have exploited the Enhanced Permeability and Retention (EPR) effect by using nano-scale drug delivery vehicles. The use of passive targeting with nano-sized drug delivery vehicles (DDV) facilitate entry into tumors via dysfunctional blood vessels and abnormal lymph nodes that enhance permeability into the tumour and enhance retention, respectively [269]. Alternatively, active targeting, in which ligand-conjugated vehicles are attached and internalized via specific receptors at the cell surface utilizing ligand-receptor interactions may be considered [270]. This ligand-based approach aims to deliver any cargo encapsulated in DDV with a high degree of precision. In this way, receptor-mediated drug delivery alters the biodistribution of the drug by increasing accumulation at the site of interest. The role of receptors as molecular targets has also provided new opportunities for cellular and intracellular targeting [266].

### 4.1. Drug Delivery Vehicles

Nanoengineered drug delivery vehicles (DDV) are designed to transport encapsulated cargo containing therapeutically active ingredients [271] to the site of action. The design of targeted DDV can be optimized by altering the size, shape, rigidity, material, ligand, and ligand orientation which contribute to cellular uptake, biodistribution, biocompatibility, drug loading and clearance [272,273,274]. DDV functionalized with ligands aim to target ion channels, enzymes, membrane carriers and receptors. It is crucial to consider other possible binding sites that may interact with DDV such as plasma proteins which may lead to clotting or immune responses that may have an impact on the efficacy of the drug delivery system [275]. A protein corona, consisting of over 50 kinds of proteins, may form around nanoparticles following administration and may influence the in vivo distribution of the nanoparticles [276] and should be investigated when designing targeted DDV [277].

#### 4.1.1. Size and Surface Charge

The benefits of nanotechnology are derived from the impact of size on biodistribution, cellular uptake and kinetics of release [278]. The main mechanisms identified in the uptake of nanoparticles are diffusion, fluid-phase endocytosis, and phagocytosis [279]. The tiny nanoparticles escape the reticuloendothelial system (RES) since macrophages fail to recognize the technology as foreign agents [280].

When evaluating nanoparticles designed for the selective delivery of a drug, the effects of particle size and surface charge on murine macrophage uptake and in vivo biodistribution demonstrated that particle size and surface charge were critical parameters. The size of nanoparticles plays a role in the manner they interact with biological systems such as the immune system for example. An investigation into the interdependent effects of physicochemical properties on cellular uptake and biodistribution showed that even relatively small physicochemical differences such as a 10-mV difference in potential changed phagocytic cell uptake. The negatively and positively charged fluorescence-labelled chitosan derivative nanoparticles evaluated displayed cell-line and energy-dependent endocytosis. The high cationic surface charge and large particle size of >100 nm resulted in higher serum protein adsorption and non-specific uptake by most cell lines when compared to that observed for neutral or anionic particles [281].

#### 4.1.2. Shape

**Figure 8:**
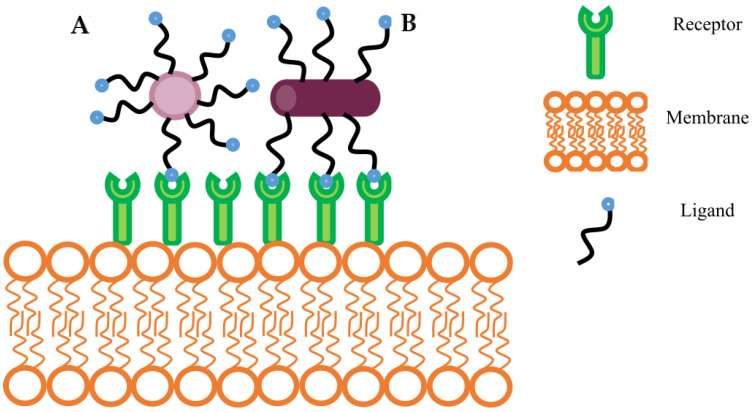
Graphical representation shows a comparison of nanocarrier shape and ligand-binding capability. (**A**) Represents a spherical shape nanocarrier. (**B**) Represents rod-shaped nanocarrier. The differently shaped nanoparticles have different active fractional area which presents variability in binding avidity [291].

Receptor-mediated endocytosis of nanocarriers is affected by shape [282]. A strategy used for targeting cells may include control of the shape of nanoparticles [283]. Nanoparticles may appear as spheres, rods, cuboidal, star-shaped, triangular, octahedral, plate-like, prisms or disk shaped [284,285,286,287,288], amongst others. Spherical nanoparticles when compared to elongated rod-shaped nanoparticles exhibit a higher aspect ratio and surface area, which facilitates multivalent interaction with the cell surfaces [289,290]. Figure 8 demonstrates differently shaped nanoparticles have different active fractional area [291]. The effect of shape on cellular uptake of gold nanoparticles and nanotriangles was investigated and cellular uptake by leukemic monocyte-macrophages in a murine model revealed gold nanorods and nanostars exhibited significantly higher uptake. An investigation into the mechanism of cellular uptake revealed that all shapes of gold nanoparticles made use of the clathrin-mediated endocytic pathway however, gold nanorod uptake was also dependent on caveolae raft-mediated endocytosis, and gold nanotriangle uptake with cytoskeletal rearrangement and the dynamin pathway [292]. The shape of nanoparticles also plays a role in margination towards the blood vessel walls following intravenous administration [293]. Nanoparticle margination is controlled by forces that influence article translational and rotational movement by electrostatic double-layer interactions, buoyancy, drag, van der Waal’s interactions, gravity, and repulsive steric interactions [291]. A study investigating the effect of size on spherical nanoparticles and the likelihood of margination, revealed that the transport of small nanoparticles exhibited greater margination than their larger counterparts [294]. Larger spherical nanoparticles movement was driven by convection, resulting in a greater difficulty in escaping the vascular flow dynamics towards the vessel wall. Rod-shaped nanoparticles exhibited a tendency to drift towards vessel walls because of drag forces and torques that are exerted on rods when in flowable systems [291].

#### 4.1.3. Elasticity

Particle elasticity is thought to alter the cellular uptake of nanoparticles through cell surface receptor binding and an ability to squeeze through pores in addition to changes in blood circulation time [295]. The intrinsic property that both hard and soft nanoparticles share is the large surface area that allows interfacial forces to dominate when in most surroundings [296]. Characterizing the interface of a nanoparticle is critical for understanding and interpreting the interaction between the nanoparticles biological system and exchange of materials with the system. Hard nanoparticles have a solid-liquid interface, whereas soft nanoparticles are characterized by a dynamic liquid-liquid interface [297]. Although more fragile, soft nanoparticles have similar surface energies to biological surfaces. Hard nanoparticles are more robust and have a higher surface tension, which may induce adverse catalytic events on the surfaces of the particle [276].

Investigation of the elasticity of nanoparticles and influence thereof on blood circulation time, phagocytosis, endocytosis and targeting properties revealed that soft particles demonstrated an enhanced circulation and enhanced targeting when compared to harder nanoparticles. In vitro experiments also revealed that soft nanoparticles demonstrated a reduced cellular uptake in immune, endothelial and cancer cells which increased persistence of soft nanoparticles in the circulatory system [298]. Sun et al. [299] used a microfluid platform to show rigidity-regulated cellular uptake of hard lipid shell rigid nanoparticles that entered cells more efficiently than flexible soft nanoparticles. To tune the rigidity of core-shell nanoparticles the interfacial water between the polymer core and lipid shell of this hybrid nanoparticle were changed, where more significant amounts of interfacial water resulted in more flexible nanoparticles [299]. Guo et al. [300] evaluated in vitro cellular uptake of nanolipogels and demonstrated that particle elasticity directly affected tumor accumulation and suggested that particle elasticity was an important design parameter for enhancing direct to tumor delivery. The core-shell structure of nanolipogels featured a lipid bilayer shell and hydrogel core that allowed modulation of elasticity by the extent of crosslinking of the hydrogel core made up of alginate. The results suggest that while soft nanoparticles entered cells by fusion and endocytosis, hard nanoparticles entered cells via clathrin-mediated endocytosis only and this mechanism of modulus-mediating cellular response can be attributed to a shift in internalization pathways from low energy fusion to high energy endocytosis [300].

#### 4.1.4. Chemical Composition

The properties of the drug to be delivered are usually the starting point when selecting materials to build a drug delivery vehicle. Biomimetic nanoparticles based on the size, hydrophobicity, and ideal drug release profile are considered vital when mimicking nature for the design of drug delivery technologies [301]. Formulation of cannabinoids is challenging due to their lipophilicity, instability to temperature changes, light and auto-oxidation [302,303]. Proteins facilitate the intracellular uptake of endocannabinoids because of their temperature-dependent properties [304,305]. Selecting a temperature-sensitive polymer may be a strategic approach to ensuring uptake of cannabinoids [306].

#### 4.1.5. Lipid-Based Drug Delivery Systems

Lipid-based drug delivery systems are a diverse formulation strategy for the incorporation, dissolving or suspension of active pharmaceutical ingredients in lipidic excipients. Lipids are esters of fatty acids such as glycerides and PEG esters comprised of hydrophobic hydrocarbon chains linked to hydrophilic, electrostatically charged head groups such as glycerides, polyglycerol, or polyalcohol esters. Classes of lipids include fatty acids, long-chain triglycerides, medium-chain triglycerides, propylene glycol esters and mono- or di-glycerides [307]. These components viz., the lipid headgroup and the hydrophobicity of the fatty acid chain, can be customized to alter the physicochemical properties or chemical functionality of the system. The length of the fatty acid chain and degree of unsaturation define the melting range, solubilization capacity and miscibility properties of the lipid excipients which have an impact on particle stability and influencing drug retention and release in the system [308].

Liposomes are composed primarily of lipids with diacyl chains and can be functionalized by conjugating targeting ligands to chemically reactive lipids. The mechanical properties of liposomes were recently investigated to determine the differences in the endocytic pathway of liposome uptake with different values for Young’s modulus [300]. Liposomes with a low modulus interacted with membranes more rapidly and efficiently than liposomes with high-modulus, which were endocytosed by the more energy consuming clathrin-mediated pathway. Liposomes have been designed to deliver a racemic mixture of a lipophilic cannabinoid type 2 agonist, MDA7 [309]. Three different systems, hydroxypropyl-β-cyclodextrins, micellar preparation, and liposomes, were formulated with MDA7 and the antiallodynic effects were compared after intravenous administration in rats.

#### 4.1.6. Polymer-Based Drug Delivery Systems

Biodegradable polymeric materials have been used to manufacture nanoparticles that cross biological barriers for the purposes of active cancer-targeting [310]. Polymeric nanoparticles can be manufactured using synthetic and natural polymers. Both approaches offer control of size and surface properties of nanoparticles however, natural polymers exhibit promising features such as biocompatibility and ready availability [311].

Micelles are formed by amphiphilic macromolecule copolymers, orientated with single acyl chains facing inward, forming an inner core and their hydrophilic headgroups facing outwardly [312]. Nano-micelles of styrene-maleic acid (SMA)-conjugated with a synthetic cannabinoid WIN-55,212 were synthesized and exhibited an increased efficacy and extensive in vivo biodistribution [313]. Poly (D, L-lactide-co-glycolide) was used [314] as a biodegradable and biocompatible colloidal carrier prepared by nanoprecipitation. The aim was to develop a nanoplatform capable of transporting the highly lipophilic active agent Δ9-THC following oral administration. To ensure enhanced uptake by intestinal cells and to minimise protein adsorption, the nanoparticle was engineered by modification of the surface with poly (ethylene glycol), chitosan, or poly (ethylene glycol)-chitosan shells.

De La Ossa et al. [315] investigated the feasibility of developing cannabinoid loaded poly-ε-caprolactone microparticles as a dosage form for the administration of the cannabinoid, cannabidiol. The polymeric drug delivery system was prepared with poly-e-caprolactone, which is biocompatible, biodegradable, and is a semi-crystalline aliphatic polyester that degrades slowly. In this way, the microspheres produced protected cannabinoids from degradation and controlled the drug release rate. Spherical microparticles of 20–50 µm with high entrapment efficiency of close to 100% were successfully produced. The results of the study showed that when compared to the free drug, the PCL microparticles provided long-term administration of cannabidiol, demonstrating the potential therapeutic advantages of the technology than when free drugs are used. Durán-Lobato [316] investigated the efficacy of three kinds of poly (DL-lactide-co-glycolide) (PLGA) nanoparticles containing synthetic cannabinoid CB13. The nanoparticles included plain PLGA nanoparticles, PLGA nanoparticles coated with polyethylene glycol (PEG) chains, and PLGA nanoparticles covalently bound with PEG chains to produce a hydrophilic surface. When compared to lipid nanoparticles, the polymeric PLGA nanocarriers exhibited an extended-release profile, were larger in size and exhibited similar high entrapment efficiency and loading capacity.

### 4.2. Surface Modification

Surface modification of nanocarriers has been used to optimize the effectiveness of targeting, improving ligand binding, and enhancing cellular uptake of the drug delivery system. The most influential factors of functionalizing the surface(s) of nanocarriers include spacing, density and orientation of ligands. It has been shown that enhanced cellular uptake and reduced nonspecific uptake in healthy tissues are attributed to the spatially distinct orientation of ligands on the surfaces of nanocarriers.

Lipid bilayers of cell membranes have been shown to form compartmentalized domains called lipid rafts or caveolae pits which exhibit different biophysical properties. The raft domain is an essential feature for membrane proteins such as G-protein coupled receptors since they provide a platform for the assembly of signaling complexes and prevent crosstalk between pathways [317]. Caveolae are a particular type of lipid raft viz., a plasma membrane invagination, that is implicated in signal transduction, transcytosis, endocytosis, and cholesterol trafficking [318]. Studies have revealed that CB1-R selected clathrin-dependent and caveolae internalization pathways equally [319]. The g-protein heterotrimeric subunits are fatty acylated proteins that target lipid rafts through fatty acylation or interactions with caveolin [320,321].

While the process of GPCR endocytosis for receptor-mediated drug delivery has been attempted [322], evidence in recent years revealed it also plays a role in receptor signaling, specifically switching the coupling of GPCR to alternative mitogenic kinase cascades [323,324].

#### 4.2.1. Ligand Density and Distribution Patterns

**Figure 9:**
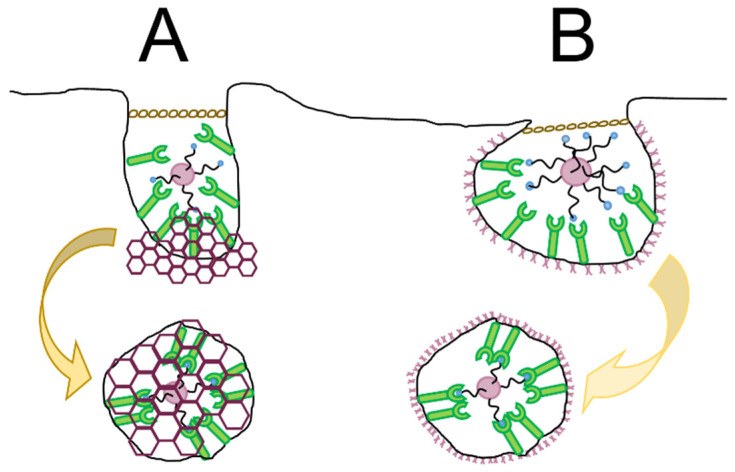
(**A**) A spherical-shaped nanoparticle with a low-density ligand pattern most likely to encounter clathrin pit for endocytosis. The clathrin-coated pit is deeply invaginated and pinched off from the plasma membrane to form a clathrin-coated vesicle following endocytosis. (**B**) A spherical-shaped nanoparticle with a high-density ligand pattern most likely enters the cell through caveolae-mediated endocytosis. The caveolae, a flask-shaped vesicle at the plasma membrane, is internalized and fused to form caveosomes. Adapted with permission from reference [326].

Studies have revealed that ligand density affects cell internalization of targeted nanoparticles and influences the pathway of nanoparticle internalization by the cells. A ligand presented in a clustered as opposed to dispersed arrangement affects and generally increases the level of cellular uptake in an in vitro epithelial model [325]. An approach to enhancing the targeting efficacy of novel drug delivery vehicles includes precise control over the spacing and density of the ligand. The potential effect(s) of ligand density and distribution patterns could have on the cellular internalization pathways such as clathrin and caveolae-mediated pathways is depicted in Figure 9. The cellular uptake of nanoparticles changes with differences in surface density of the ligand, where high ligand density systems are taken into cells effectively via the caveolae-mediated pathway, and low surface ligand density systems are internalized less efficiently through the clathrin-mediated pathway [325].

Ligand-specific endocytic dwell times of CB1-R are able to be used to control the functional selectivity of the receptor. The amount of time during which CB1-Rs are clustered into clathrin-pits with β-arrestins prior to endocytosis was found to be the mechanism by which β-arrestin signaling was controlled [327]. Agonists capable of modulating the amount of time CB1-R interacted with β-arrestins in the endocytic pit were identified and WIN 55,212-2 elicited a short endocytic dwell time and activated mainly G-proteins, whereas the endogenous 2-AG agonist exhibited a prolonged dwell time with short term G-protein and longer-term β-arrestin signaling. It was postulated that endocytic agonists induce specific phosphorylation patterns in the intracellular domain of the CB1-R which is thought to stabilize specific conformations of the receptor that promote differential interaction with β-arrestins at the plasma membrane while also initiating signaling [234,328].

#### 4.2.2. Dual, Asymmetric, and Clustered Ligands

A multitude of ligands can be conjugated with the surface of nanovehicles and may perform multiple roles including targeting receptor-mediated cellular uptake of CB1-R or CB2-R agonists or antagonists, natural macromolecules, and cell-penetrating peptides. When ligands attached to the surface of nanoparticles in a traditional manner the surface pattern is random across the nanoparticle surface. It was demonstrated [329] in a study to manipulate ligands in specific groupings to control the cluster group size and spacing that the efficacy and specificity of targeted delivery using ligand conjugated nanoparticles was enhanced by introduction of a dendritic design for a polymer nanovector that created clustered or patchy ligand features on micellar surfaces [329]. Patchy and ligand clustered micelles exhibited promise with higher binding affinities and minimal off-target cytotoxicity [330]. The ligand arrangement on nanoparticle rotation during the adhesion and engulfment processes provided a theoretical foundation for patchy nanoparticle design, which contribute to an improved ligand design for drug delivery [331].

## 5. Discussion and Future Perspectives

Subsequent to the discovery of endocannabinoid receptors, the complex endocannabinoid system consists of bioactive lipidic endocannabinoids, their synthesizing and degrading enzymes, the endocannabinoid membrane transporter and the endocannabinoid receptors, research has revealed that the ECS regulates neurotransmitter release, brain development, tissue and organ homeostasis, synaptic plasticity and cytokine release and it follows that the dysregulation of the ECS can be observed in multiple pathophysiological disorders [330]. Decades of research has also changed our early view of the ECS and initiated optimism for global legalization of one of the most well studied phytocannabinoids, *Cannabis Sativa* [330]. The side effects of high Δ9-THC content which includes paranoia, psychosis, impaired memory, and motor coordination and altered judgement in short-term use and addiction, cognitive impairment, chronic lung inflammation and increased risk of chronic psychosis disorders in long-term use, deters patients from making use of the therapeutic benefits the plant extract may offer [330]. The serious adverse health events resulting in deaths by highly potent CB1-R synthetic cannabinoids AMB-FUBINACA and FAAH inhibitor BIA 10-2474 prompted a deeper focus on understanding the ECS which lead to an exploration of novel strategies including biased signaling, allosterism, and peripheral restriction [330]. A myriad of opportunities for specific targeting of the endocannabinoid system exists. Box 1 summarizes the main strategies for specific targeting in this article. We suggest nanotechnology as a promising tool to utilize the strategies outlined.

Specific targeting of the endocannabinoid system seems to be a good starting point towards developing a sophisticated cannabinoid drug design void of undesirable side effects but the future of commercialized ECS products calls for exploration from a broader perspective. Further study into the complexity of the expanded endocannabinoidome is required to consider the dynamics and interconnections it has with other regulatory systems. As the ECS is interconnected with other lipid-based signaling systems and cannabinomimetic compounds have been identified in a variety of foods, research into the link between diet and the synthesis and release of endocannabinoids and related mediators will do well to guide a better understanding of the endocannabinoidome and epigenetics of the ECS [330].

Box 1 **The biologic and pharmacological understanding of cannabinoid receptors, their expression and biodistribution can be used to achieve specific pharmacological outcomes at target sites.**

   **Novel strategies for targeting the endocannabinoid system**

- The full characterization of signaling pathways is essential when considering candidates for targeting the ECS.
- Approaches to activate either G-protein signaling or β-arrestin signaling, include the use of endocytic lipid rafts, binding at specific extracellular loop motifs and plasticity of the receptor.
- Methods for using routes of biased signaling, include designing ligands as structural tools including allosteric modulators or ligands that exploit the plasticity of the receptor as G-protein biased ligands or β-arrestin biased ligands.
- High-resolution crvstal structures of cannabinoid receptors and molecular simulations effectively guide cannabinoid drug design.
- Nanoparticles can be engineered to provide site-specific delivery of cannabinoid ligands.
- Altering the density and surface pattern of ligands through clustering and patterns is a strategy to promote the use of one route of endocytosis over another.
- Overexpression of receptors in different pathological states can be used as potential targets for treatment of specific disorders.
- Receptor-mediated endocytosis can also be used to facilitate entry into specific sites through cannabinoid receptors.
- Molecular simulations can be used to predict the motion of every atom in a receptor and those in the molecules with which the receptor interacts.

This box summarizes the key points contained in the article.

## Figures and Tables

**Table 1:** The location and intensity of CB1 receptors in the human body.

Intensity	Location	Reference
	Brainstem cardiorespiratory centers	[10]
Low		
Moderate	Spinal cord	[114]
Dense	Hippocampus	[115]
	Basal Ganglia (globus pallidus, substantia nigra)	[116]
	Cerebral cortex (cingulate gyrus and prefrontal cortex)	[117]
	Amygdala	
	Cerebellum	
	Ventral horn	[118]
	Hypothalamus	

**Table 2:** The location and intensity of CB2 receptors in the human body.

Intensity	Location	Reference
	Bones	[84]
Low	Spleen	[123]
	Tonsils	
	Mast cells	
	Blood cells	
Moderate	Skin	[124]
Dense	Neuron	[125]
	Human lung	[69]
	Uterus	
	Thymus	
	Pancreas	
	Reproductive tissues	

## Data Availability

Not applicable.

## Abbreviations

CBD	Cannabidiol
ECS	Endocannabinoid system
EPR	Enhanced permeation and retention
PEG	Polyethylene glycol
MAGL	Monoacylglycerol lipase
FAAH	Fatty acid amide hydrolase
Δ9-THC	(−)-Δ9-tetrahydrocannabinol
NAPE-PLD	*N*-acyl phosphatidylethanolamine-selective phospholipase D
DAGL	*sn*-1-selective diacylglycerol lipase
2-AG	2-arachidonoylglycerol
EMT	endocannabinoid membrane transporter
COX-1	*Cyclooxygenase type 1*
COX-2	*Cyclooxygenase type 2*
FABP5	Fatty acid-binding proteins
DDV	Drug delivery vehicle
